# Humanization interventions in primary health care: an umbrella review

**DOI:** 10.3389/frhs.2026.1835535

**Published:** 2026-08-10

**Authors:** Augusto Mellado, Eric Tapia Escobar, Sebastián Pavlovic, Marcela Riveros, Diego Silva-Jiménez

**Affiliations:** 1 Facultad de Medicina y Ciencias de la Salud, Universidad Central de Chile, Santiago, Chile; 2 Facultad de Medicina y Salud, Universidad Finis Terrae, Santiago, Chile

**Keywords:** humanization in health, primary health care, patient-centered care (PCC), person-centered care, shared decision-making (SDM), empathy, care experience, umbrella review

## Abstract

**Introduction:**

This umbrella review identified systematic and non-systematic reviews of quantitative and qualitative empirical studies on interventions aimed at promoting humanization in Primary Health Care (PHC). The objective of this umbrella review was to synthesize and compare the available evidence on interventions aimed at promoting humanization in PHC. It addressed two questions: (1) What are the main definitions and dimensions of humanization in healthcare identified in the existing body of research? and (2) Which interventions promote humanization in PHC, and what outcomes have they demonstrated?

**Methods:**

The reviews were selected following PRISMA guidelines and eligibility criteria based on the PICOS strategy (population, interventions, comparators, outcomes, and study design). Records were identified in Web of Science (WoS), Scopus, and PubMed.

**Results:**

Twenty-five systematic and non-systematic reviews were included. The evidence shows consistent convergence in defining humanization in PHC as person-centered and relational care grounded in open communication, empathy, respect, and patient participation in decision-making. The most frequently studied interventions include person-centered communication styles, shared decision-making, reorganization of care processes (e.g., welcoming practices, continuity, and coordination), and organizational strategies targeting access and equity. These interventions are robustly associated with improvements in experiential outcomes, such as satisfaction, trust, participation, reduced decisional conflict, and strengthened therapeutic relationships, while effects on final clinical outcomes are more variable and context-dependent. Community-based, intercultural, and digital interventions were also identified, extending humanization toward social determinants, continuity of care, and reduced access barriers.

**Discussion:**

Findings showed that humanization in PHC aligns with classic Person-Centered Care frameworks, where patients' values, preferences, and life contexts guide clinical decisions. Relational micro-practices emerge as central quality mechanisms, yet their impact depends on enabling organizational conditions, including time, continuity, coordination, and institutional support. Overall, humanization appears as a system-level property rather than an isolated technique, requiring integrated, context-sensitive, and equity-oriented interventions to achieve sustained and meaningful effects.

## 1 Introduction

In recent years, there has been a growing interest in the humanization of healthcare as a domain as relevant as the technical and economic dimensions that underpin health systems. This interest appears to reflect an increasing awareness of the inherent limitations of healthcare models that, in practice, remain predominantly focused on technical and biomedical aspects (1–3), despite longstanding critiques advocating for the integration of biological, psychological, social, and even technical dimensions (4–6). While scientific and technological advances have profoundly transformed the diagnosis and treatment of disease, it has become increasingly evident that such progress alone is insufficient to ensure effective and satisfactory care. Within this context, humanization emerges as a necessary approach to balance technical-clinical developments with a more comprehensive understanding of the human experience of suffering, illness, and the relational needs inherent to human beings (7, 8).

Humanized care has increasingly consolidated as a key trend within healthcare systems, driven by the recognition of patients not merely as carriers of disease, but as whole persons with histories, values, beliefs, emotions, and social relationships (9, 10). While medical specialization and the fragmentation of knowledge have been fundamental to clinical advancement, they have also contributed to creating emotional and communicative distance between professionals and patients, thereby obscuring the human bond that should underlie any act of care. In this sense, humanization should not be understood as an optional complement, but rather as an essential component of comprehensive and high-quality care. The aspirations of healthcare systems are ultimately oriented toward greater justice, equity, and respect for persons; therefore, humanization constitutes an ethical imperative that requires ongoing reflection and the continuous development of evidence to support its practical implementation (11–13).

Achieving humanized care may require the development of a set of personal competencies that enable healthcare professionals to carry out their work with respect for human dignity and rights, emphasizing a holistic notion of well-being. Among the dimensions proposed to address humanization in healthcare are: (a) affect, defined as the capacity to appropriately process emotional information and to empathize with another person's affective state without conflating one's own feelings with those of others; (b) self-efficacy, referring to confidence in one's ability to successfully manage complex and stressful situations in the workplace; (c) emotional understanding, understood as the ability to comprehend, identify, and cognitively interpret the emotions and feelings of others; (d) optimistic disposition, the tendency to maintain positive expectations regarding future events; and (e) sociability, characterized by a preference for interacting with others and the ability to relate appropriately through assertiveness and empathy (14). These dimensions are grounded in specific ethical and bioethical principles that place human dignity, empathy, and respect for autonomy at the center of care. From a relational perspective, there is a recognized need to foster respectful and supportive relationships among patients, professionals, and institutions, promoting practices that integrate the values of care, justice, non-maleficence, and the protection of the most vulnerable (15).

Two recent systematic reviews of international studies addressing the humanization of healthcare across different health systems highlight key aspects that help to elucidate its practical implementation. The first review reports that the relational domain was the most extensively explored and discussed across the 20 studies it included [2007–2016], emphasizing: (i) relational support for patients and their caregivers within the context of a genuine and sensitive relationship; (ii) empathy and respect for dignity, uniqueness, individuality, and humanity; (iii) consideration of all biopsychosocial dimensions of patients, illness, and care; (iv) respect for patient autonomy, participation, and the fulfillment of their needs and expectations; and (v) the competence and psychological preparedness of healthcare professionals to understand patients (16). In turn, the second review focused on healthcare services in Ibero-America, encompassing 30 studies and reviews of various types [2008–2017], and highlighted: (i) the safeguarding of human dignity across all stages of the life course, particularly at its beginning and end; (ii) the centrality of care provided by others, recognizing human beings as inherently interdependent; and (iii) communication, empathy, and respect for patients' rights, wishes, and preferences regarding the care of their own health (17).

The assessment of some of these dimensions has been carried out primarily within the field of nursing, with at least three instruments developed and validated in Spanish-speaking populations. The first includes the dimensions of respect (prudence, respect for life, respect for autonomy, and truthfulness), responsibility (personalized care, proximity, and empathy), quality (quality, competence, and knowledge), and transpersonal care (compassion, simplicity, and diligence) (18). The second instrument conceptualizes humanization as a multidimensional construct composed of five factors: affect, self-efficacy (professional competence), emotional understanding (empathy), optimistic disposition, and sociability (14). Finally, the third instrument identifies a four-factor model consisting of humanization, non-infantilization, respect, and empowerment (19).

One approach to establishing procedures that foster humanization in healthcare has been through the framework of Patient-Centered Care (PCC), which seeks to strengthen the relationship between the healthcare system and patients by prioritizing individual needs, values, and preferences across all aspects of care (20, 21). PCC is widely recognized as a fundamental pillar of healthcare quality, emphasizing the relationship between healthcare professionals, the patient, and their family. This approach promotes shared decision-making (SDM), with careful attention to patients' values and needs, as well as their empowerment through access to information that is clear, reliable, understandable, and emotionally meaningful (22).

On the other hand, several studies highlight the importance of Primary Health Care (PHC) as a fundamental pillar for achieving more accessible and equitable health systems, capable of balancing medical and social dimensions in order to respond effectively to patients' needs (23). These studies emphasize the importance of listening to patients' voices and incorporating their values (such as satisfaction with care, preferences, priorities, and experiences within the healthcare system) into health-related decision-making processes. In particular, taking patients' values into account may improve access to care, continuity of care, and adherence to treatment. However, a number of challenges hinder the achievement of these goals. Long working hours and the emotional demands placed on healthcare workers require increasing levels of resilience in contexts characterized by interpersonal relationships marked by suffering, vulnerability, deterioration, and illness. The high psychological burden associated with healthcare work contributes to professional burnout and negatively affects providers' own health, as expectations extend beyond physical capabilities to include significant psychological demands (24).

In this regard, more specific selection processes may be required for PHC professionals, given that this level of care entails particular competencies distinct from those required in other healthcare settings. The selection of professionals suited to these demands could contribute to more effective practices, alongside the need to provide ongoing training for those already in service in order to promote behavioral change and improvements in management (25). Additional challenges in PHC include dissatisfaction with the physical infrastructure and equipment of healthcare services, as well as with patient flow processes, all of which may either facilitate or hinder access to care. These issues are compounded by shortages of healthcare professionals, fragmentation of work processes, and ambiguities in professional roles and responsibilities. At the same time, there is a need to underscore the importance of relational technologies (such as reception, bonding, active listening, respect, and dialogue) which are essential for professional–patient interactions and cannot be replaced by so-called “hard” technologies (26).

Other models, such as SDM and social prescribing (SP), propose complementary and convergent strategies for the humanization of healthcare by reinforcing individuals' active role in their care processes (27, 28). Systematic training of primary care professionals has been shown to enhance knowledge, communication skills, clinical confidence, and patients' effective participation in decision-making, thereby reducing paternalistic practices and fostering decisions more closely aligned with patients' values and preferences. SP, in turn, demonstrates that social referrals are most effective when grounded in trusting relationships, personalized interventions, and sustained support. These approaches activate mechanisms such as self-efficacy, empowerment, and motivation, while also mobilizing community and territorial resources.

In Latin America, one of the countries that has most systematically implemented humanization in healthcare as part of its national policy framework is Brazil. In this context, humanization has been promoted through the Unified Health System (SUS), with the establishment of the National Program for the Humanization of Hospital Care (PNHAH) in 2000, followed by the National Humanization Policy (PNH), also known as HumanizaSUS, in 2003. This policy is grounded in principles of transversality, the inseparability of care and management, and the active participation of individuals in healthcare processes, with PHC serving as the central axis for its universal implementation (29).

Finally, humanization in healthcare, consistently supported by research, demonstrates that effective, patient-centered communication not only has a positive impact on patients' mental and physical health, promoting well-being and improved recovery, but also enhances satisfaction, participation in decision-making, and adherence to treatment. Ultimately, recognizing and respecting patients' rights as fundamental human rights is essential to safeguarding their dignity and integrity within the global healthcare experience (30, 31).

Although there is a growing body of theoretical contributions and empirical studies supporting the use of humanized practices in healthcare, there remains a lack of systematized evidence regarding the specific interventions that can produce measurable outcomes within health systems, particularly in PHC, which constitutes the main point of entry into the healthcare system in most Western countries. Furthermore, the large number of reviews available in this area highlights the relevance of conducting an umbrella review to provide a comprehensive overview of the current state of the scientific evidence, thereby supporting decision-making by policymakers, healthcare professionals, and researchers.

Consequently, the objective of this umbrella review was to synthesize and compare the available evidence on interventions aimed at promoting humanization in PHC. This review of the specialized literature seeks to address the following questions: (1) What are the main definitions and dimensions of humanization in healthcare identified in the existing body of research? and (2) Which interventions promote humanization in PHC, and what outcomes have they demonstrated?

## 2 Materials and methods

In this review, the Preferred Reporting Items for Systematic Reviews and Meta-Analyzes (PRISMA) guidelines (32, 33) were used, and the PICOS (participants, interventions, comparators, outcomes, and study design) strategy was used to establish the eligibility criteria for the articles (34).

The search was conducted in high-quality databases, including the Web of Science Core Collection (WoS), SCOPUS, and PubMed, the latter selected for its specialization in and comprehensive coverage of scientific articles in medicine, health, psychology, nursing, and biomedical sciences. The search strings were as follows: WoS: TS = (human* OR “people-centered” OR “person-centered” OR “patient-centered” OR “family-centered”) AND TS = (“primary care” OR “primary healthcare” OR “family medicine”) NOT TS = (“ICU” OR “Hospital” OR “Palliative”); SCOPUS: TITLE-ABS-KEY [(“humanization” OR “people-centered” OR “person-centered” OR “patient-centered” OR “family-centered”) AND (“primary care” OR “primary healthcare” OR “family medicine”) AND NOT (“ICU” OR “Hospital” OR “Palliative”)] AND [LIMIT-TO (DOCTYPE, “re”)]; and PubMed: (“humanization” OR “people-centered” OR “person-centered” OR “patient-centered” OR “family-centered”) AND (“primary care” OR “primary healthcare” OR “family medicine”) NOT (“ICU” OR “Hospital” OR “Palliative”), with no time restrictions applied. Although the term “humanization” is widely used in some regions, particularly in Latin America and parts of Europe, the international literature often describes similar concepts using alternative terms such as Person-Centered care or PCC. Data extraction was conducted on January 30, 2025 (WoS and SCOPUS) and on June 26, 2025 (PubMed).

According to the checklist of the PRISMA guidelines (32, 33), the following quality steps for systematic reviews were verified according to thesesections: 1 (title), 2 (structured abstract), 3 (rationale), 4 (objectives), 5 (eligibility criteria), 6 (sources of information), 7 (search strategy), 8 (selection process), 9 (data extraction process), 10a and 10b (data items), 16a and 16b (study selection), 17 (study characteristics), 19 (results of individual studies), 23 (discussion), 25 (support), 26 (competing interests), and 27 (availability of data, code and other materials). The following sections were excluded because the data from each study to satisfy their criteria were not considered pertinent within the narrative synthesis of the present umbrella review, or were not available, or were presented only in a general way after having been part of a respective protocol: 11 (study risk of bias assessment), 12 (effect measures), 13 (synthesis methods), 14 (reporting bias assessment), 15 (certainty assessment), 18 (risk of bias in studies), 20 (results of syntheses), 21 (reporting biases), 22 (certainty of evidence), and 24 (registration and protocol).

Through PRISMA guidelines, the selection of articles was specified based on eligibility criteria: the target population (participants), the interventions (methodological techniques), the elements of comparison of these studies, the outcomes of these studies, and the study designs (the criteria of the PICOS strategy as shown in Table 1). The selection of the included reviews was conducted by the five authors of this study. Initially, each abstract was independently screened, and only those accepted by at least two reviewers were included in the subsequent phase. In the next stage, each author evaluated a subset of full-text reviews and proposed those deemed suitable for inclusion in the final phase. Finally, authors AM, ET, and SP reached consensus on the reviews to be included in the analysis. The deduplication and collaborative screening process was supported through the use of the Rayyan tool (35), ensuring transparency, reproducibility, and inter-reviewer consensus at each stage of the selection process. A formal methodological quality appraisal of the included reviews was not undertaken because the primary objective of this umbrella review was to provide a comprehensive synthesis and comparison of the humanization interventions reported in the review literature, rather than to assess the certainty of the evidence or generate specific technical recommendations. Nevertheless, methodological rigor was promoted through an independent review process conducted by pairs of researchers, with discrepancies resolved through discussion and consensus. In addition, the review was carried out by an interdisciplinary team affiliated with an academic unit specializing in humanization in health care, contributing subject-matter expertise to the screening, selection, and interpretation of the included evidence.

**Table 1:** Eligibility criteria using PICOS (participants, interventions, comparators, outcomes, and study design) strategy.

PICOS	Description
P (Participants)	Patients, healthcare professionals (e.g., physicians, nurses, social workers), and administrative staff in primary care centres
I (Interventions)	Any intervention aimed at improving the humanization of primary care, such as: - Training in clinician–patient communication. - Patient-centred care models. - Respect and dignity programmes. - Technological and non-technological interventions.
C (Comparators)	Usual (standard) care. No specific intervention.
O (Outcomes)	Humanization indicators:
	- Patient satisfaction. - Quality of staff–patient communication. - Perceived respect and dignity. - Improvements in patient-centred care. - Overall quality of care.
S (Study design)	Systematic reviews, empirical reviews, and meta-analyses based on: - Randomized controlled trials (RCTs). - Quasi-experimental studies. - Observational studies (cohort, case-control). - Qualitative studies.

## 3 Results

**Figure 1:**
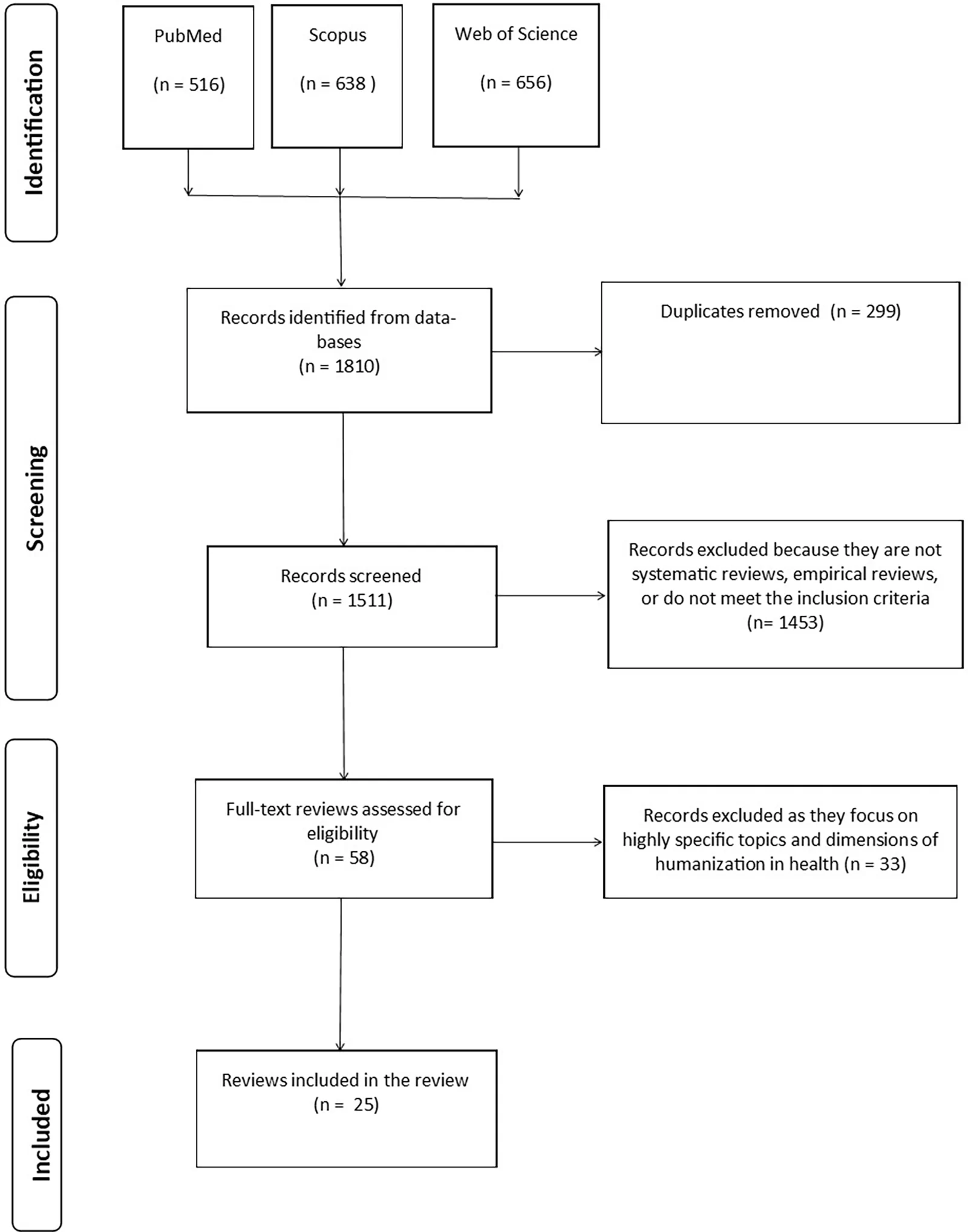
PRISMA—flow diagram.

In the initial stage of the review selection process, a total of 1,810 records were identified through systematic searches across the aforementioned databases: Web of Science (*n* = 656), Scopus (*n* = 638), and PubMed (*n* = 516). After the removal of 299 duplicates, 1,511 unique records were screened based on titles and abstracts. During this screening phase, 1,453 records were excluded for not constituting systematic or empirical reviews, or for failing to meet the predefined inclusion criteria. As a result, 58 articles were assessed in full text to determine their eligibility. At this stage, 33 reviews were excluded because their focus was limited to highly specific dimensions of humanization in healthcare (e.g., highly specialized clinical contexts, or outcomes not transferable to the primary care level). Ultimately, 25 reviews met all inclusion criteria and were incorporated into the present umbrella review (see flow diagram in Figure 1).

From a descriptive standpoint, the 25 reviews included in this study reflect a heterogeneous empirical base, encompassing systematic reviews (8 reviews), systematic reviews with meta-analyses (2 reviews), qualitative meta-syntheses (1 review), integrative reviews (4 reviews), literature reviews (2 reviews), systematic hermeneutic reviews (1 review), scoping (5 reviews) and realist reviews (2 reviews). The included reviews were published between 2008 and 2024.

Regarding geographical contexts, the reviews frequently draw on primary studies conducted in high-income countries (e.g., the United States, the United Kingdom, Canada, Australia, and European countries), particularly when addressing communication, SDM, care coordination, and digital innovations in PHC (36–41). At the same time, a substantial body of Latin American evidence, particularly from Brazil, frames humanization as an institutional policy and practice linked to access, reception/welcoming processes, and community orientation, as well as a response to inequities in the organization of care at the primary level of the health system (26, 42, 43).

In terms of participants and methods, the evidence synthesized in the reviews includes patients/users, PHC professionals, and multiprofessional teams, as well as, in several interventions, liaison or support roles (e.g., patient navigators, community health workers, and link workers in SP) (44–47). Methodologically, the studies encompassed quantitative, qualitative, and mixed-methods designs, particularly when examining experiences and mechanisms of change, with controlled trials (including RCTs) reported in structured interventions targeting multimorbidity or decision-making (36, 48). A summary of the general characteristics of the included systematic reviews is presented in Table 2.

**Table 2:** Characteristics of the included reviews.

ID	Authors and year	Journal	Journal Impact Factor (Best quartile)	Authors' countries	Countries of the reviewed studies	Number of documents reviewed and type of studies	Title	Method
1	Charlton et al. (2008) (37)	Journal of the American Academy of Nurse Practitioners	JCR 1.6/Q2	United States	United States	7 studies (quantitative empirical studies, mostly descriptive, some experimental and randomized)	Nurse practitioners' communication styles and their impact on patient outcomes: an integrated literature review	Integrated literature review; narrative synthesis
2	Simpson et al. (2022) (55)	BMC Health Services Research	JCR 2.9/Q1	United States	United States	20 studies (observational case studies, pre/post, quasi-experimental, and qualitative studies)	How health systems facilitate PCC and care coordination: a case series analysis to identify best practices	Case series analysis based on a rapid realist review; comparative cross-case synthesis
3	Arantes et al. (2016) (42)	Ciência & Saúde Coletiva	JCR 2.117/Q3	Brazil	Brazil	98 articles (45% qualitative, 26% quantitative, 8% mixed methods, 11% reviews)	The benefits and challenges of the Family Health Strategy in Brazilian Primary Health care: a literature review	Literature review across three dimensions: policy/institutional, organizational, and technical/care-related
4	Sharma et al. (2017) (52)	BMC Health Services Research	JCR 2.6/Q1	United States	United Kingdom (15), United States (8), Canada (4), Australia/New Zealand (4), Sweden (1)	32 studies (1 RCT, 4 quasi-experimental studies, 2 systematic reviews, 1 cross-sectional survey, 9 qualitative/ethnographic studies, 16 case studies)	The impact of patient advisors on healthcare outcomes: a systematic review	Systematic review; PRISMA; qualitative thematic synthesis
5	Hughes et al. (2020) (56)	The Milbank Quarterly	JCR 7.8/Q1	United Kingdom	Australia, Canada, Italy, the Netherlands, New Zealand, Sweden, the United Kingdom, the United States, Denmark	71 documents (31 primary studies, 22 evidence reviews, 14 theoretical/conceptual reviews, 4 policy documents)	Rethinking Integrated Care: A Systematic Hermeneutic Review of the Literature on Integrated Care Strategies and Concepts	Systematic hermeneutic review with iterative analysis of empirical, conceptual, and policy literature
6	Teggart et al. (2023) (57)	BMC Health Services Research	JCR 2.9/Q1	Canada	United States, United Kingdom, Canada, the Netherlands, Sweden, China, Greece, Croatia, Spain	21 studies (8 RCTs [38%], 7 one-group pre–post studies [33%], 6 non-randomized two-group studies [29%])	Effectiveness of system navigation programs linking primary care with community-based health and social services: a systematic review.	Systematic review; PRISMA; narrative synthesis
7	Bailey et al. (2021) (58)	Journal of Medical Internet Research (JMIR)	JCR 5.4/Q1	United States	United States	41 studies (pragmatic randomized controlled trials [pRCTs])	Early Patient-Centered Outcomes Research Experience With the Use of Telehealth to Address Disparities: Scoping Review	Scoping review (Arksey & O’Malley); PRISMA-ScR; Active Implementation Framework; narrative synthesis and iterative Delphi
8	Frost et al. (2015) (50)	Journal of Primary Care & Community Health	JCR 2.1/Q2	Australia	Australia; United Kingdom; the Netherlands; Poland; Croatia; Japan; China; Canada; Belgium; Germany; Sweden; Switzerland	24 documents (2 literature reviews, 17 quantitative studies, 3 qualitative studies, and 2 mixed-methods studies)	An Integrative Review of Enablement in Primary Health Care	Narrative integrative review (Whittemore & Knafl); narrative synthesis
9	Nora and Junges (2013) (26)	Revista de Saúde Pública	JCR 3.1/Q1	Brazil	Brazil	40 studies (32 scientific articles, 2 theses, 4 dissertations, and 2 book chapters, all qualitative research)	Humanization policy in primary health care: a systematic review	Systematic review with qualitative meta-synthesis; interpretive synthesis
10	Mitre et al. (2012) (43)	Ciência & Saúde Coletiva	JCR 1.6/Q2	Brazil	Brazil	28 articles (qualitative studies [89%], quantitative and mixed-methods)	Avanços e desafios do acolhimento na operacionalização e qualificação do Sistema Único de Saúde na Atenção Primária: um resgate da produção bibliográfica do Brasil [Advances and Challenges of Acolhimento (Welcoming Practices) in the Operationalization and Improvement of the Unified Health System in Primary Care: A Review of the Brazilian Literature]	Narrative literature review; critical-reflective review
11	Percival et al. (2022) (47)	Family Medicine and Community Health	JCR 4.3/Q1	Canada	United Kingdom and South Korea	7 studies (quantitative studies, mainly before–after [pre–post] without a control group; 1 quality-improvement study with a comparison group)	Systematic review of SP and older adults: where to from here?	Systematic review (PRISMA), Synthesis Without Meta-analysis(SWiM), and quality appraisal using the NIH tool for pre–post studies without a control group
12	Poitras et al. (2018) (54)	BMC Health Services Research	JCR 2.6/Q1	Canada	United States; Canada; United Kingdom; the Netherlands	4 systematic reviews (quantitative and qualitative studies)	What are the effective elements in patient-centered and multimorbidity care? A scoping review	Scoping review (Arksey & O’Malley); narrative synthesis and mapping of intervention elements associated with positive health outcomes
13	Ahmed et al. (2022) (38)	Health & Social Care in the Community	JCR 2.9/Q1	Netherlands	Countries in Europe and North America	55 documents (systematic reviews, scoping reviews, reviews of reviews, meta-analyses, empirical studies, guidelines, reports, and grey literature)	Person-Centered Care in primary care: What works for whom, how and in what circumstances?	Rapid realist review; Realist And Meta-narrative Evidence Syntheses: Evolving Standards (RAMESES)
14	Khatri et al. (2023) (39)	BMC Primary Care	JCR 2.4/Q2	Australia; Nepal; Ethiopia	United States, Canada, the Netherlands, United Kingdom, Australia, Uganda, Nigeria, South Africa	52 documents (reviews [*n* = 9], quantitative studies [*n* = 5], qualitative studies [*n* = 36], and mixed-methods [*n* = 2])	People-centred primary health care: a scoping review	Scoping review; PRISMA-ScR; thematic analysis using the Framework Method (Gale et al.) and synthesis organized according to the five World Health Organization—Framework on Integrated People-Centred Health Services (WHO-IPCHS) components
15	Smith et al. (2021) (48)	BMC Systematic Review	JCR 5.1/Q1	Ireland; Canada	Germany; United Kingdom; United States; Canada; Ireland; Spain; Australia	23 RCTs (16 studies included in the narrative synthesis/7 included in the meta-analysis)	Interventions for improving outcomes in patients with multimorbidity in primary care and community settings: a systematic review	Systematic review; PRISMA; update of a Cochrane review; Grading of Recommendations Assessment, Development and Evaluation (GRADE)
16	Peart et al. (2018) (44)	BMJ Open	JCR 2.3/Q2	Australia	United States	20 documents (programme descriptions, intervention studies, retrospective studies)	Patient navigators facilitating access to primary care: a scoping review	Scoping review (Arksey & O’Malley; Levac et al.); descriptive synthesis and content analysis (Freeman framework)
17	Prajankett and Markaki (2021) (45)	International Nursing Review	JCR 2.1/Q2	United States; Thailand	United States; Thailand	42 documents (systematic reviews, quantitative, qualitative, and observational studies)	Integrated older people care and advanced practice nursing: an evidence-based review	Integrative review; PRISMA; Melnyk & Fineout-Overholt hierarchy of evidence; inductive synthesis
18	Cooper et al. (2023) (46)	Perspectives in Public Health	JCR 3.0/Q2	United Kingdom	United Kingdom	6 studies (qualitative and mixed-methods studies)	Service user perspectives on SP services for mental health in the UK: a systematic review	Systematic review; PRISMA; thematic synthesis of qualitative data (Thomas & Harden)
19	Leonardsen et al. (2023) (41)	Healthcare	JCR 3.1/Q2	Norway; Sweden	United States; Sweden; France; Iceland; the Netherlands; New Zealand; Norway; United Kingdom	12 studies (RCTs, qualitative studies, and quantitative intervention studies)	Person-Centeredness in Digital Primary Healthcare Services—A Scoping Review	Scoping review (Arksey & O’Malley); PRISMA-ScR; thematic synthesis
20	Dahm et al. (2022) (51)	Journal of General Internal Medicine	JCR 3.3/Q1	Australia and United States	United States, United Kingdom, the Netherlands, Switzerland, Finland, Sweden	19 studies (6 quantitative, 3 mixed-methods, and 10 qualitative studies)	Communication of Diagnostic Uncertainty in Primary Care and Its Impact on Patient Experience: an Integrative Systematic Review	Integrative systematic review: PRISMA; thematic and content synthesis
21	Zulman et al. (2020) (49)	JAMA	JCR 45.5/Q1	United States	Not reported	73 studies (RCTs and controlled observational studies. The review was complemented by 27 clinical observations, 67 qualitative interviews, and a modified Delphi with 14 experts)	Practices to Foster Physician Presence and Connection With Patients in the Clinical Encounter	Mixed-methods study integrating: (1) systematic literature review, (2) rapid ethnographic observations, (3) qualitative interviews with healthcare professionals, patients, and non-medical professionals, and (4) a modified Delphi for consensus; narrative synthesis
22	Kunneman et al. (2019) (40)	Patient Education and Counseling	JCR 3.6/Q1	United States; the Netherlands; Puerto Rico	Countries in Europe, North America, Asia, Oceania, and Latin America	14 studies (prospective descriptive studies and controlled trials [interventional and non-interventional])	Humanistic communication in the evaluation of SDM: A systematic review	Systematic review; PRISMA; content analysis
23	Scalia et al. (2018) (53)	Patient Education and Counseling	JCR 3.6/Q1	United States; the Netherlands	United States; United Kingdom; Germany; Canada; Australia; Greece; Spain; Belgium; Italy; China; the Netherlands	53 studies (23 RCTs in the meta-analysis and 30 non-randomized studies [qualitative, observational, pre–post, and quasi-experimental] in the narrative synthesis)	The impact and utility of encounter patient decision aids: systematic review, meta-analysis and narrative synthesis	Systematic review: PRISMA; meta-analysis and narrative synthesis
24	Stacey et al. (2011) (36)	Cochrane Database of Systematic Reviews	JCR 8.0/Q1	Canada; United States; United Kingdom	Australia; Canada; China; Finland; Germany; the Netherlands; United Kingdom; United States	86 RCTs	Decision aids for people facing health treatment or screening decisions	Systematic review (Cochrane and PRISMA); meta-analysis using random-effects models
25	Cáceres Rivera et al. (2024) (59)	Public Health	JCR 4.1/Q1	Colombia; Spain	Australia; Colombia; United Kingdom; Argentina; Brazil; Canada; Chile; Ecuador; Spain; Honduras; United States	28 documents (systematic reviews, qualitative studies, quantitative studies, mixed-methods studies, guidelines, and policy documents).	Parameters for delivering ethnically and gender-sensitive primary care in cardiovascular health through telehealth. Systematic review	Systematic review (PRISMA and Cochrane); qualitative narrative synthesis

The narrative synthesis of definitions and dimensions of humanization suggests that it is commonly conceptualized as a person-centred and relational form of care, in which the clinical encounter is structured through open, comprehensible, and bidirectional communication that integrates patients' beliefs and social context, in contrast to more directive biomedical styles (37, 38, 40). Within this framework, empathy, respect, and active listening are consistently identified as core dimensions, alongside practices aimed at sustaining clinical presence and fostering meaningful connection, including the explicit acknowledgement of patients' interests (38, 40, 49). Moreover, several reviews indicate that humanization is reflected in experiential outcomes such as patient satisfaction, trust, and treatment adherence, which are associated with patient-centred communication styles and the validation of illness experiences (37, 48, 50). Complementarily, it is emphasized that humanizing care also entails communicating complex situations with honesty (such as diagnostic uncertainty), while preserving participation, informed consent, and the therapeutic relationship even in the absence of clinical certainty (40, 49, 51).

Another key aspect is that humanization in healthcare is conceptualized in terms of patient, family, and community agency and shared responsibility, such that care aligns with individuals' values, preferences, and life goals, rather than being driven solely by biomedical indicators (26, 39, 52). Within this framework, SDM emerges as a central dimension. Emphasis is placed on the provision of clear information regarding options, risks, and benefits; the clarification of values; and the reduction of decisional conflict in order to support informed decisions that are consistent with what matters to the person (36, 40, 53). Consistently, the literature also indicates that humanization requires relational and organizational conditions that render it feasible such as sufficient consultation time, clear communication, verification of understanding, and individualized care planning, particularly in contexts of multimorbidity and high complexity (38, 48, 54). Furthermore, participation extends beyond the clinical encounter through co-design and advisory mechanisms (e.g., patient advisory councils), which have an impact on safety, educational materials, institutional priorities, and the overall care experience (26, 52, 55). A substantial body of reviews also incorporates the concept of person-centred care, understood as organizing healthcare not solely around disease, but around the integration of patients' values, preferences, life context, autonomy, and capabilities, through a clinical relationship grounded in communication, respect, SDM, and shared responsibility in care (38, 39, 41, 45, 46, 48, 54, 56, 57).

In organizational terms, the reviews converge in indicating that humanization cannot be reduced to individual competencies; rather, it depends on continuity, coordination, and integration of care, supported by structures, incentives, and institutional values that enable person-centred practices (38, 55, 56). Drawing particularly on the experience of the Brazilian health system and its national humanization policy, this approach is linked to the democratic reorganization of work, acolhimento (welcoming practices), a structured welcoming and reception practice in care, the fostering of therapeutic bonds, and the inseparability of care and management, incorporating communicative transversalism and spaces for social participation (26, 42, 43). In reviews grounded in integrated care and people-centred care frameworks, key dimensions include governance and accountability, participatory models of care, intersectoral coordination, and enabling environments (including financing), thus framing humanization as a property of the health system as a whole (39, 45, 55). Finally, strategies such as system navigation and patient navigation operationalize humanization by reducing barriers, providing guidance and accompaniment, coordinating referrals, and facilitating effective access particularly for vulnerable populations and at the interface between healthcare, social services, and community resources (44, 56, 57).

Across the literature, several reviews incorporate equity as a constitutive dimension of humanization. In this sense, to humanize care entails identifying and reducing structural barriers (social, cultural, administrative, geographical, or technological) that constrain access and continuity, and adapting care to conditions of vulnerability and diversity (44, 57, 58). In digital and telehealth contexts, humanization is reframed as technology-mediated person-centredness, with particular emphasis on accessibility, patient empowerment and self-management, and situational awareness, alongside emerging tensions related to the risk of depersonalization when digital tools replace rather than facilitate the therapeutic relationship (41, 55, 58). Within this same perspective, explicit parameters are proposed for care that is responsive to ethnicity and gender, including cultural competence, intercultural mediation, self-determination, and community participation, with a focus on differential access and quality, particularly in telehealth settings (38, 58, 59). Finally, community embeddedness emerges as a humanizing dimension when primary care integrates non-medical resources through SP, promoting a sense of belonging, social reconnection, autonomy, non-stigmatizing care, and context-sensitive responses to psychosocial needs (39, 46, 47). A summary of the definition of humanization, dimensions of humanization studied, types of healthcare institutions examined, and participants in the studies in each of the reviews can be found in Table 3.

**Table 3:** Definition of humanization, dimensions of humanization studied, types of healthcare institutions examined, and participants in the studies in each of the reviews.

ID	Authors and year	Definition of humanization in healthcare	Dimensions of humanization studied	Types of institutions examined	Participants
1	Charlton et al. (2008) (37)	Patient-centred communication/biopsychosocial style: a Nurse Practitioner (NP)–patient interaction that actively incorporates the patient, uses open-ended questions, encourages mutual participation, and considers ideas/beliefs, emotions, and social context, in contrast to a more directive/authoritarian biomedical style focused on signs and symptoms.	(a) Patient satisfaction/approval, (b) adherence to treatment plans, and (c) health/improvement (including self-reports and some markers/measures). Communication barriers (time, discomfort, knowledge/memory/hearing/vision deficits, etc.) are also described as conditions shaping humanization understood as an effective communicative relationship.	Settings in which NPs act as providers (mainly primary/ambulatory care) and patient outcomes are discussed.	NPs and patients. Reported sample sizes across studies range from *n* = 48 to *n* = 1,316.
2	Simpson et al. (2022) (55)	Integrated, continuous care responsive to patients' values and needs, emphasizing patient experience, effective communication, and institutional accountability.	(a) Institutional values; (b) patient–provider and provider–provider communication; (c) use of technological infrastructure (IT) to facilitate human interactions; (d) accountability and incentive mechanisms; (e) organizational structures supporting intervention implementation.	Health systems and high-performing organizations.	Patients, healthcare professionals, and authorities within the health systems analyzed.
3	Arantes et al. (42)	Acolhimento (welcoming and reception practices), therapeutic relationship, multiprofessional work, community orientation, family focus, and person-centred practices.	(a) Technical/care-related and (b) policy/institutional (equity, participation).	Family health units, municipal services, and community experiences.	Healthcare professionals (physicians, nurses, dentists, community workers), managers, students, service users, and families.
4	Sharma et al. (2017) (52)	Active engagement of patients, families, and caregivers in improving healthcare through a joint partnership.	(a) Active patient participation in organizational decision-making; (b) impact of patient advisory councils on clinical outcomes; (c) patient safety and satisfaction; (d) impact on educational materials and physical spaces; (e) psychosocial benefits for patient advisers themselves.	Primary care centres, hospitals, specialty clinics, emergency services, outpatient care, and mental health institutions.	Patients and family members serving as advisers (advisory councils, committees, co-design groups, community panels), along with the clinical and administrative staff linked to those councils.
5	Hughes et al. (2020) (56)	Person-centred and coordinated care that enables patients to participate in planning, experience continuity, receive effective communication, and obtain psychosocial support.	(a) Control, planning, communication, and emotional and practical support for patients; (b) coordination across services; (c) recognition of the person as an active subject rather than a passive object.	Health centres.	Patients with multimorbidity and their caregivers; healthcare professionals in multidisciplinary teams; and policy-makers.
6	Teggart et al. (2023) (57)	Integrated, Person-Centered Care that reduces barriers and facilitates continuous, effective, and efficient access.	(a) Equitable and continuous access to services; (b) person-/patient-centred approach; (c) integrated care across health and social services; (d) reduction of structural and social inequities; (e) support for caregivers and families.	System navigation programmes in primary healthcare linked to community institutions and organizations (social services, mental health, housing, chronic disease support, etc.).	Adults aged ≥18 using primary care, including people with chronic illness or chronic risk factors, and in some cases their caregivers.
7	Bailey et al. (2021) (58)	Reduction of barriers and attention to populations at risk of inequities.	(a) Technological access (internet, devices, connectivity); (b) geographic access and transportation; (c) provider availability; (d) support for self-care, education, and patient–team communication.	Outpatient institutions. Primary and/or community care.	Patients at risk of disparities, including racial/ethnic minorities, people in rural areas, low-income communities, individuals with low health literacy, and people with disabilities.
8	Frost et al. (2015) (50)	Enablement is understood as an outcome of the consultation that helps people cope with life and health problems. It is associated with the therapeutic relationship, seeing the person as a whole, facilitating learning, valuing strengths, supporting decision-making, and expanding possibilities.	(a) Therapeutic relationship; (b) open communication and patient-centred style; (c) trust, hope, and legitimization of illness; (d) autonomy, coping, and independence; (e) influence of social and cultural factors (ethnicity, socioeconomic situation, health status).	Mainly PHC, focused on general practitioners' consultations, but also including some nursing and tertiary-sector studies.	Patients with chronic diseases (asthma, diabetes) Patients from different ethnicities and contexts Healthcare professionals: GP and nurses.
9	Nora and Junges (2013)(26)	Concrete social and professional practices that transform ways of acting and caring, in line with the National Humanization Policy (PNH/HumanizaSUS).	(a) Transversality (greater communication among subjects and services); (b) inseparability of care and management (clinical-political articulation); (c) autonomy, co-responsibility, and agency of subjects and collectives.	Mainly Primary Care Units within Brazil’s Unified Health System (SUS), including health centres, Family Health Strategy programmes, and local PHC services.	Users (patients and families), health professionals (physicians, nurses, community workers), and in some cases managers.
10	Mitre et al. (2012) (43)	Ensuring universal and equitable access, with professionals sensitive to the needs of users and communities, by reorganizing the healthcare work process in a democratic and reflective manner. Its operational axis is humanized reception (*acolhimento*).	(a) Timely and universal access to PHC; (b) responsiveness to health demands; (c) sensitivity and bonding among professionals, users, and communities; (d) overcoming the hegemonic biomedical model; (e) democratic spaces for participation and reflection; (f) team training and capacity-building.	pPHC Units and the Family Health Programme.	Health professionals (physicians, nurses, PHC teams). Community health workers. Users and communities served in PHC.
11	Percival et al. (2022) (47)	A SP model understood as a person-centred and relationship-centred approach that seeks to respond to non-medical social needs, strengthen community ties, and personalize care in PHC. Humanization is expressed as social reconnection, meaningful participation, and care that goes beyond the biomedical model.	(a) Psychosocial (well-being, loneliness, social participation, self-esteem, self-efficacy), (b) physical (physical activity, frailty), and (c) use of health resources (general practioner [GP] visits, costs). Also includes a relational-community dimension.	PHC centres, community services, cultural centres, and social and community programmes linked to PHC.	Older adults (≥60 years), predominantly women. In some studies, family caregivers also participated.
12	Poitras et al. (2018) (54)	A patient-centred care framework applied to multimorbidity: care that promotes SDM, a holistic view of the person (beyond individual diseases), adaptation to social and family context, and reorganization of services to reduce treatment burden and fragmentation. Humanization is understood as reorienting the system and clinical practice toward the person in context.	(a) Relational-clinical (SDM, communication, continuity); (b) personal (self-management, empowerment, quality of life); (c) organizational (case/care management, interdisciplinary work, use of IT); (d) professional (training and competencies).	Primary healthcare in systems with a strong primary-care base. Includes PHC clinics, integrated community programmes, and organizational models of chronic care.	Adults with chronic illnesses and multimorbidity (mostly ≥50 years). Health teams (physicians, nurses, care managers, interdisciplinary teams) as part of the intervention apparatus.
13	Ahmed et al. (2022) (38)	Defined as Person-centered Care, incorporating understandable communication, respect, empathy, SDM, and personalized care plans. Humanization is understood as a coherent relational and organizational practice, not as an isolated technique.	Integrated multilevel dimensions: (a) relational-communicative (empathy, clear language, listening, therapeutic relationship); (b) personal (self-management, involvement, concordance); (c) organizational (accessibility, consultation time, coordination, teamwork); (d) systemic/policy (training, supportive policies).	Primary healthcare (general practice, primary care services) and health systems. Includes clinical practice, PHC organizations, and links with other domains (social, community).	PHC patients, with explicit emphasis on people with low health literacy and ethnic and socioeconomic diversity. Healthcare professionals (GPs, nurses, interdisciplinary teams) also participate as key PCC agents.
14	Khatri et al. (2023) (39)	Conceptualized as people-centred PHC within the WHO -IPCHS framework. It entails placing people and communities—not diseases—at the centre of the system, promoting holistic care, respect, empathy, participation, co-responsibility, and continuity across the life course. Humanization is understood as a relational, organizational, and systemic transformation of PHC.	Multilevel dimensions organized according to the five IPCHS components: (a) engagement and empowerment (empathy, participation, co-production); (b) governance and accountability; (c) model of care (participatory, residential, multimorbidity-focused); (d) intersectoral coordination; (e) enabling environment and financing.	Primary healthcare and primary care. Includes PHC centres, primary care clinics, Patient-Centred Medical Home-type models, community services, and integrated care arrangements.	PHC users (adults, older adults, people with multimorbidity), families and communities, and healthcare professionals (interdisciplinary teams, managers, organizational leaders).
15	Smith et al. (2021) (48)	Interventions for multimorbidity that prioritize patient experience, care coordination, self-management, person-centred communication, and treatment optimization, aligned with the principles of person-centred care.	(a) Patient experience (satisfaction); (b) relational-communicative (continuity, empathy, priority-focused communication); (c) self-management; (d) organizational (coordination, case management); (e) pharmacological (medication appropriateness and optimization).	Primary care and community settings. PHC clinics, general practice consultations, interdisciplinary teams, home care, clinical pharmacies.	Adults with multimorbidity (mostly ≥65 years, with ≥3 conditions). PHC teams (GPs, nurses, pharmacists, social workers, occupational therapists, psychologists) participate as intervention agents.
16	Peart et al. (2018) (44)	A relational support process that facilitates effective access to primary healthcare through patient navigators, especially for vulnerable populations, by providing information, support, barrier reduction, and care coordination.	(a) Relational-communicative (bond-building, accompaniment, trust); (b) patient experience (information, understanding of the system, respectful treatment); (c) organizational (integration of fragmented systems, PHC—emergency departments—community coordination); (d) equity and access (reduction of social, cultural, and administrative barriers).	Primary healthcare, emergency departments, hospital services, community clinics, and community-based settings.	People without a regular PHC provider, mostly vulnerable populations: uninsured individuals, people living in poverty, migrants, people with mental illness, taxi drivers, and people with chronic illness. Navigators included community health workers, lay workers, volunteers, and case managers.
17	Prajankett and Markaki (2021)(45)	Explicitly defined through the WHO- IPCHS framework applied to the care of older people. It implies integrated, Person-Centered Care responsive to needs, preferences, and context, with emphasis on empowerment, coordination, continuity, governance, and enabling environments. Humanization is understood as a systemic property of care.	Multilevel dimensions organized according to the five IPCHS strategies: (a) engagement and empowerment (empathy, participation, co-production); (b) governance and accountability; (c) model of care (participatory, residential, multimorbidity-focused); (d) intersectoral coordination; (e) enabling environment and financing.	Primary care and community care for older adults: PHC clinics, home care, long-term care, ambulatory clinics, and community services.	Older adults (≥60 years), families, and informal caregivers. APNs as central agents, in addition to community teams and volunteers.
18	Cooper et al. (2023) (46)	Person-centred, relational, and non-stigmatizing care that recognizes psychosocial suffering, promotes autonomy, social belonging, and meaningful support beyond the biomedical model.	(a) Supportive relationship with the link worker (trust, listening, validation); (b) subjective care experience (feeling understood and respected); (c) autonomy and agency; (d) belonging and social connection; (e) emotional safety and non-stigmatization.	PHC and the community sector, GP practices, SP services, voluntary and community organizations, and cultural and social activities linked to PHC.	Adult users of SP services with mental health difficulties (anxiety, depression, psychosocial distress). Link workers participate as key agents.
19	Leonardsen et al. (2023) (41)	Conceptualized as PCC applied to digital PHC services. It implies care that recognizes the person as an active agent, promotes participation, autonomy, understanding of life circumstances, and adaptation of care to the individual context, even when interaction is mediated by technology.	(1) Accessibility (to information, interaction, and services); (2) self-management and empowerment; (3) tensions between digitalization and person-centredness (risk of depersonalization); (4) situation awareness (contextual understanding of the patient by professionals and by the patient themself).	Primary healthcare and digital community care. Includes GP practices, community PHC, home care, and digital services associated with PHC.	Adults and older adults (≥45 years) with chronic conditions (chronic obstructive pulmonary disease, congestive heart failure, diabetes, cancer, etc.), caregivers, and PHC professionals (nurses, coaches, physicians, healthcare staff).
20	Dahm et al. (2022) (51)	Humanized care is centred on how diagnostic uncertainty is communicated to the patient: it involves making explicit that there is no definitive explanation, involving the person in the diagnostic process, sustaining SDM and informing them of risks and follow-up plans. Humanization is linked to patient-centred, empathic, and honest communication that seeks to preserve trust, participation, and informed consent even in the absence of diagnostic certainty.	(a) Professional–patient relationship and clinical communication (verbal and non-verbal expressions of uncertainty, empathy, humour, expectation management); (b) technical-care dimension linked to diagnostic reasoning (making the diagnostic process explicit, use of differentials, exclusion of serious diagnoses); (c) patient participation in decisions and experience of care (trust, satisfaction, anxiety, adherence); (d) ethical aspects of honesty about uncertainty and its relationship to diagnostic error and informed consent.	PHC settings: general/family medicine, general internal medicine, and general pediatrics in PHC clinics, university health centres, and hospital outpatient services.	Primary care physicians (family physicians, general internists, pediatricians, residents, and in some studies medical students), together with adult and pediatric patients and, in some studies, parents and caregivers. The aggregated total is 839 physicians and 6,037 patients across the 19 included studies.
21	Zulman et al. (2020) (49)	Addressed through the concept of “clinical presence”: a deliberate practice of awareness, focus, and attention aimed at understanding and connecting with the patient. Interpersonal interaction is understood as a source of personal and contextual data (what matters to the patient, how illness affects their life and goals), enabling the construction of respect and trust while reducing errors and misunderstandings. Humanization is threatened by time constraints, intrusive electronic records, and administrative demands, and a set of concrete practices is proposed to restore meaningful connection in the clinical encounter.	(a) Therapeutic relationship and communication (active listening, verbal and non-verbal language, use of silence, body posture); (b) participation and shared responsibility (joint agenda setting, agreement on “what matters most” to the patient, SDM); (c) emotional/affective dimension (exploration and validation of emotions, empathy, recognition of emotional cues); (d) consideration of the patient's life context (life story, social and cultural circumstances, patient efforts and small achievements); (e) organizational/care process dimension (impact of time, the Electronic Health Record, and organizational culture on the capacity for presence and connection).	Three primary care clinics, an academic medical centre, a Veterans Affairs facility, and a federally qualified health center.	Primary care physicians (10 in the observational phase; 8 in the Delphi panel, in addition to other clinical experts). Adult patients (27 observed/interviewed, English- and Spanish-speaking, with diverse sociodemographic backgrounds). Professionals from other highly relational fields (30 interviewed: management, business/finance, community and social services, education, arts/media, protective services, and personal care). Health system leaders, patient advocates and caregivers, and clinical communication researchers also participated in the Delphi panel.
22	Kunneman et al. (2019) (40)	Humanistic communication is understood as the manner and content of the clinician–patient interaction that expresses dignity, respect, active listening, empathy, and concern for the patient's human situation; it includes attention to emotional, social, and practical aspects in addition to technical ones.	(a) Empathy/therapeutic relationship; (b) respect and listening; (c) psychosocial support; (d) socio-emotional vs. technical communication; (e) provision of information.	Mainly primary and secondary care: primary care, oncology, cardiology, surgery, geriatrics, pediatrics, rehabilitation services, and intermediate hospital settings; the review spans studies across multiple levels of care.	Patients/users (including parents in pediatrics), healthcare professionals (physicians/clinicians), external observers (coders), and in some studies families/caregivers. Instruments included patient self-report, clinician report, and observational measures.
23	Scalia et al. (2018) (53)	Facilitation of SDM, increased knowledge, and reduction of decisional conflict.	(a) Therapeutic relationship/communication and participation in decisions; (b) technical-care dimension (knowledge, risk comprehension, decision scales); (c) organizational dimension (integration into workflows, time, training); (d) barriers/facilitators (time, training, format/content).	PHC, hospitals/specialty services (cardiology, oncology, psychiatry), outpatient services, emergency care, and musculoskeletal clinics.	Patients/users (including caregivers/parents), clinical professionals (physicians, nurses, others), teams, and managers involved in implementation.
24	Stacey et al. (2011) (36)	Active participation in clinical decisions involving uncertain benefits/harms, provision of information on options and outcomes, and achievement of informed decisions congruent with what the person values.	(a) Therapeutic relationship/communication (effects on patient–professional communication); (b) participation and autonomy in decision-making; (c) values clarity; (d) experience/satisfaction and decisional conflict; (e) behaviour (proportion undecided, choice); (f) health outcomes, costs, and consultation time.	PHC unspecified (trials across multiple clinical contexts and varied decisions; the review does not delimit level of care).	Patients facing treatment or screening decisions; in some cases, decisions for children or significant others lacking decision-making capacity.
25	Cáceres Rivera et al. (2024) (59)	Ethnic- and gender-sensitive approach: holistic and flexible care, accessible services, continuous quality improvement, a culturally competent workforce, self-determination, and community participation; also differential access to resources, women's health, intercultural mediation, and sexual orientation.	(a) Relationship/communication; (b) access and equity; (c) community participation and co-responsibility (self-determination, participation, community agents); (d) technical-care dimension (operational parameters, telemonitoring, coordination); (e) policy/institutional dimension (regulatory framework, guidelines); (f) physical/organizational environment (appropriate infrastructure, information systems).	Community and PHC settings predominate; interventions and guidelines applicable to PHC centres, community services, and national regulatory bodies.	Users/patients (Indigenous peoples and women), community agents and Indigenous health workers, PHC professionals, multidisciplinary teams, and also policy-makers and public bodies through regulations.

Reviews consistently indicate that interventions targeting person-centred communication styles constitute one of the most robust axes of humanization in primary healthcare. These interventions include the systematic use of open-ended questions, active listening, exploration of patients' beliefs and concerns, incorporation of the psychosocial context, and the explicit promotion of patient participation in clinical decision-making. The synthesized evidence shows consistent associations between these communication styles and higher patient satisfaction, improved treatment adherence, and better self-reported health outcomes, compared to traditional biomedical approaches, without necessarily requiring substantial increases in consultation time (36, 37, 55).

Another set of interventions focuses on SDM, for example through the use of patient decision aids in primary care (36, 53). These interventions aim to improve patients' knowledge, enhance their understanding of risks and benefits, clarify values, and reduce decisional conflict. Reviews report robust effects in terms of increased knowledge, greater observed participation, reduced decisional conflict, and higher satisfaction, although evidence remains less conclusive regarding changes in clinical outcomes or long-term adherence. From a humanization perspective, these interventions reinforce the recognition of the patient as a moral and decision-making agent, even though their implementation often faces organizational barriers such as perceived time constraints or insufficient professional training (36, 40).

Organizational and management interventions emerge as a third central axis for humanization in primary healthcare, particularly in reviews from Latin American contexts. These include the implementation of acolhimento, the reorganization of care flows, co-management involving both staff and service users, the establishment of humanization groups, and practices such as home visits, qualified listening, and individualized therapeutic plans. Reported outcomes include improved access, reduced waiting times, strengthened therapeutic relationships, and increased patient trust and satisfaction. However, the literature also identifies tensions related to resource constraints, professional resistance, and challenges in integrating humanization as a structural dimension of management rather than as an isolated practice (26, 42, 43).

Similarly, some reviews highlight interventions aimed at care coordination and continuity, embedded within Person-Centered Care frameworks. These strategies include the use of optimized electronic health records, interdisciplinary teamwork, population health programmes, telemonitoring, and continuous quality improvement cycles. The synthesized evidence indicates improvements in patient experience, perceived quality of care, clinical communication, and institutional accountability, underscoring that humanization depends critically on organizational infrastructures, alignment of institutional values, and adequate technological support (55, 56).

Finally, a set of interventions is identified with a strong emphasis on equity, as well as community-based and intercultural approaches, particularly in cardiovascular primary care and in contexts involving Indigenous populations and women. These include telehealth, intercultural mediation, the integration of community health workers, local capacity-building, and cost-effective strategies to reduce barriers to access. The findings indicate greater acceptability, improved continuity of care, reduced travel burden, and a potential decrease in health inequities, although challenges persist related to digital divides, insufficient cultural competence, and the continued dominance of biomedical approaches. From a humanization perspective, these interventions broaden the focus from the individual clinical encounter to the social, cultural, and territorial determinants shaping the experience of care (57, 59). A summary of type, objectives, characteristics, assessment tools, period, and main findings of interventions implemented in each of the reviews can be found in Table 4.

**Table 4:** Type, objectives, characteristics, assessment tools, period, and main findings of interventions implemented.

ID	Authors and year	Number and type of interventions	Objectives/intervention areas	Characteristics/components of the interventions	Intervention assessment tools	Intervention period	Intervention outcomes
1	Charlton, et al. (2008) (37)	Two main types of interventions: (1) biomedical communication style and (2) biopsychosocial/patient-centred communication style delivered by NPs.	To improve patient outcomes through more participatory communication styles; increase satisfaction, adherence, and health outcomes; assess the impact of the NP's autonomous role in care/NP–patient clinical communication.	Use of open-ended questions, active listening, exploration of patients' beliefs and concerns, inclusion of the psychosocial context, patient participation in decisions, and a climate of respect and collaboration.	Patient satisfaction questionnaires, adherence scales (self-report), health-status measures (patient-reported and/or clinical indicators), clinical records, and post-care surveys.	No standardized intervention periods were reported. Assessments were conducted during routine clinical care or at short- to medium-term follow-up (weeks to months).	Patient-centred communication styles were consistently associated with greater satisfaction, better adherence, and better health outcomes.
2	Simpson et al. (2022) (55)	Person-Centered Care and Care Coordination (CC)/3 PCC/CC interventions per case, 12 documented interventions in total.	To improve patient experience and continuity and coordination of care; strengthen communication among providers and with patients; increase clinical efficiency; implement organizational and technological structures to sustain PCC/CC.	Use of optimized electronic records; payment models that incentivize accountability; interdisciplinary team-based care; population health programmes; technology-based communication channels; organizational bodies supporting implementation.	Hospital Consumer Assessment of Healthcare Providers and Systems (HCAHPS), Clinician and Group Consumer Assessment of Healthcare Providers and Systems (CGCAHPS), clinical indicators (readmissions, wait times, diagnostic accuracy), electronic health record (EHR) metrics, Interactive Voice Response (IVR) telemonitoring, and Plan-Do-Study-Act (PDSA) improvement cycles.	Not specified.	PCC and CC improved quality metrics, patient experience, and clinical communication; increased institutional accountability; facilitated large-scale implementation; and showed that technological infrastructure and alignment of institutional values are critical for sustaining change.
3	Arantes et al. (2016) (42)	Interventions within the Family Health Strategy (FHS) were not specified.	To strengthen PHC through the FHS in terms of access and equity, system organization, community participation, comprehensiveness of care, humanization and bonding, health prevention and promotion, and workforce training and management.	Formation of multiprofessional teams (physicians, nurses, dentists, community workers), home visits, community orientation, family focus, health promotion and prevention actions, coordination of the service network, and social participation.	Focus groups, interviews, ethnographic observation, cross-sectional surveys, epidemiological indicators, clinical records, and institutional evaluations.	Not specified.	Improvements in access, coverage, and equity; treatment adherence; strengthening of professional–user bonding; greater comprehensiveness and coordination of care; and progress in maternal-child health and chronic disease control.
4	Sharma et al. (2017) (52)	Patient-inclusive councils: 11 Patient Advisory Councils; 4 Community Councils; 8 *ad hoc* Committees (for a specific topic or project); 4 experience-based co-design projects; and other formats (e.g., mental health service-user groups).	To improve clinical quality. Patient safety. Patient satisfaction/experience. Set care priorities. Create educational materials and redesign spaces. Empower patients and generate psychosocial benefits.	Regular meetings of patients and family members with clinical staff. Co-design of processes and services. Creation of educational materials. Changes in visiting hours, physical spaces, and care flows. Translation of health messages into community language. Inclusion in hospital and academic committees.	Satisfaction and experience surveys. Pre–post evaluations of clinical outcomes (e.g., hypertension control, inhaler use, cancer screening). Interviews and focus groups.	One-off projects and permanent programmes.	Improved intention to undergo chronic disease screening. Reported reductions in medical errors (up to 62%). Increased satisfaction.Reduced complaints. Psychosocial benefits (empowerment, reduced stigma, skill development). Greater alignment of care priorities with the Patient-Centered Medical Home (PCMH) model.
5	Hughes et al. (2020) (56)	12 Case Management 8 Multidisciplinary Working 14 Linking Organizations 12 Whole-System Approaches	To reduce avoidable hospitalizations and improve continuity of care. To promote collaborative work for patients with complex needs. To overcome fragmentation across levels (hospital–community). To reorganize systems for chronic care and older adults.	Case management programmes (Evercare). Interdisciplinary teams with shared protocols. Clinical pathways and inter-institutional agreements. National reforms and integrated models (Chronic Care Model, National Health Service (NHS) Vanguards).	Patient experience and satisfaction surveys. Service utilization measures (hospital admissions, emergency visits). Economic and cost-effectiveness evaluations (Evercare model, NHS evaluations). Qualitative analyses (patient narratives, professional interviews).	Programmes of varying duration.	Improvements in patient and professional satisfaction. Partial reduction in hospitalizations. Better perceived continuity and coordination. Limited evidence for cost reduction or long-term sustainability.
6	Teggart et al. (2023) (57)	21 PHC interventions across 4 models: lay person-led (*n* = 10), health professional-led (*n* = 4), team-based (*n* = 6), and self-navigation supported by non-clinical staff (*n* = 1). Navigators are individuals who help patients and their families navigate the health and social care system, reducing barriers and facilitating access to services.	To integrate PHC with community health and social services in order to improve utilization and access/patient experience, health behaviours, activation/empowerment.	Navigator training (from 3 h online to a 16-week course), liaison and referral roles, support/advocacy, coaching and goal-setting, personalized lists through EMR (HealtheRx), multidisciplinary teams (nurses, social workers, CHWs, link workers).	Service utilization: administrative/insurance records (PHC visits, hospitalizations, emergency care, home care). Patient experience: Consumer Assessment of Healthcare Providers and Systems—Patient-Centered Medical Home (CAHPS-PCMH), Patient Assessment of Chronic Illness Care (PACIC), Canadian Institute for Health Information indicators (CIHI) indicators. Quality of life/health: SF-12/SF-36. Activation/self-efficacy: varied measures depending on the study.	As needed (53%), monthly (24%), weekly (12%), bimonthly (6%), one-time only (6%).	Some lay-led models reduced emergency visits and hospitalizations, while others did not. Team-based models showed more appropriate service use. Health behaviours yielded mixed results. Activation/empowerment showed specific improvements (e.g., HealtheRx increased confidence in finding resources). Patient experience improved consistently in lay-led and professional-led models. Social access and costs were rarely assessed.
7	Bailey et al. (2021) (58)	41 telehealth interventions (all pragmatic RCTs). Mobile devices 29/41 (71%) Web-based 30/41 (73%) Real-time videoconferencing 15/41 (37%) Remote monitoring 8/41 (20%) Store-and-forward 4/41 (10%)	Health monitoring Access to specialized care Health education Self-management of chronic diseases	Patient-centred design Cultural and linguistic adaptation Trusted intermediaries (community workers, lay health workers) Additional human clinical support Partnerships with payers for reimbursement	Electronic health records (EHR) Patient and advisor surveys Qualitative interviews with patients and stakeholders Remote-monitoring data (wearables, apps, home devices)	Variable duration, from 1 to 5 years.	High satisfaction in interventions using culturally adapted motivational messages. Increased knowledge with culturally adapted apps. Better adherence with messages in native languages and support from patient navigators. Greater self-management and confidence among Indigenous patients. Overall, telehealth was effective in improving access, satisfaction, and some clinical indicators, but depended on technical support, cultural adaptation, and financial sustainability.
8	Frost et al. (2015) (50)	Longer consultation duration, promotion of open communication, strengthening physician empathy, continuity of care, and validation of the Patient Enablement Instrument (PEI) as an improvement tool.	To improve patient enablement in primary care, understood as the capacity to cope with health problems, gain confidence, autonomy, and self-care.	Longer and more patient-centred consultations; empathic and clear communication style; recognition and legitimization of illness experience; continuity of care; support in decision-making; patient empowerment.	Mainly the PEI; also the Consultation and Relational Empathy (CARE) measure and questionnaires on quality of life, empowerment, and patient satisfaction.	Variable duration.	Interventions aimed at improving enablement showed a consistent association with longer consultations, a patient-centred communication style, professional empathy, continuity of care, and specific interventions for chronic diseases.
9	Nora and Junges (2013) (26)	Multiple “devices” and practices reported in the included qualitative studies: reception, bonding, home visits, qualified listening, humanization work groups, ombudsman services, shared/co-management, individualized therapeutic projects, reception classification, and worker training programmes.	To improve access and flow of care (queues, waiting times, referrals), strengthen work processes (profile and number of professionals, fragmentation), enhance relational technologies (welcoming, listening, respect, dialogue), and promote participation and co-responsibility.	Organizational interventions (signage, access circuits, referral/counter-referral), management interventions (co-management, work groups, ombudsman, continuing education), and clinical-relational interventions (humanized reception, home visits, qualified listening, Individual Therapeutic Project).	Semi-structured interviews, questionnaires, focus groups, workshops, group discussions, participant observation, and document analysis.	Not specified.	Findings indicate that humanization practices improve quality of care and user satisfaction, achieving greater bonding/trust, welcoming, and satisfaction in specific experiences. However, challenges were also identified, such as lack of resources, resistance among some professionals, and the need for better integration of the policy into health service management.
10	Mitre et al. (2012) (43)	Restructuring work processes in PHC units. Inclusion of acolhimento as a participatory management tool, with reorganization of care flows and task distribution in multiprofessional teams. Implementation of humanized reception as a professional stance in receiving users and qualified listening. Experiences of individual humanized reception and, more recently, collective acolhimento, incorporating users into the construction of therapeutic projects. Use of humanized reception as a strategy for bonding, accountability, and shared responsibility between professionals and communities. In-service training processes and reflective spaces for professionals. Continuing education as a strategy to raise awareness about humanization and move beyond a reduced notion of “humanized triage”.	To expand access to PHC. To reorganize the healthcare work process. To overcome the biomedical model. To introduce democratic, participatory, and user-centred practices.	Humanized reception (*acolhimento*) as an operational guideline, tool, or strategy. Multiprofessional team and shared responsibility. Risk and prioritization protocols. Territorialization of care. Inclusion of users in management. Adaptation of physical and environmental spaces, and use of information technologies.	Interviews, qualitative analyses, perceptions of professionals/users, and case studies in PHC services.	Not specified.	Implementation of humanized reception increased access; sensitized professionals; introduced protocols that improved prioritization; adapted waiting rooms; included users in therapeutic projects; and increased electronic record-keeping. Challenges included persistence of biomedical hegemony, workload overload, difficulties integrating PHC with specialized levels, rigidity in protocol use, precarious working conditions, lack of training, lack of democratic spaces, and bureaucratization through ICTs.
11	Percival et al. (2022) (47)	Intervention model: SP. Includes multiple heterogeneous community interventions (arts-based, physical activity, social groups, museums, gardens, etc.) linked through referral from PHC.	To reduce social isolation, improve psychosocial well-being, increase social participation, and potentially moderate healthcare resource use among older adults.	(1) Referral from PHC (GP or others); (2) in some cases, a link worker; (3) non-medical community activities; (4) focus on individual needs and preferences; (5) variable and weakly standardized implementation.	Warwick–Edinburgh Mental Well-being Scale (WEMWBS), Short Warwick–Edinburgh Mental Well-being Scale (SWEMWBS), Korean version of the Geriatric Depression Scale (GDS-K), UCLA Loneliness Scale, Rosenberg Self-Esteem Scale, EuroQol 5-Dimension, 5-Level Questionnaire (EQ-5D-5L), Museum Wellbeing Measure for Older Adults, GP visit records, and costs.	Short- to medium-term interventions: between 10 weeks and 12 months, with pre–post measurements. Limited follow-up and high variability.	Heterogeneous but generally positive outcomes in well-being and social participation. Mixed results regarding healthcare resource use. Variable adherence and high dropout rates.
12	Poitras et al. (2018) (54)	Seven types of interventions grouped into three broad categories: (a) patient-oriented: Person-Centered Care, SDM, holistic view of the patient, adaptation to context); regular and continuous clinical follow-up; individualized interventions; inclusion of family and caregivers; support for self-management (education, skill development, empowerment); (b) professional: training in patient-centred care; multimorbidity management training; development of the care/case manager role; feedback and supervision; training for interdisciplinary work; and (c) organizational: case/care management; interdisciplinary work; care coordination; support for evidence-based decision-making; and use of technologies (Electronic Medical Records [EMR], reminders, telehealth).	To achieve positive health outcomes through patient-centred and multimorbidity interventions/decision-making, self-management, care coordination, teamwork, professional training, and technological support.	Regular clinical follow-up, individualized interventions, consideration of family/caregivers, education and self-management support, case/care management, interdisciplinarity, continuing professional training, use of Information and Communication Technologies (ICTs) (EMR, reminders, telehealth).	Results derive from multiple clinical, psychosocial, and quality-of-life instruments used in the original studies (not standardized or comparable across studies).	Highly variable periods: from brief interventions (weeks) to prolonged follow-up (months or indefinite), depending on the programme and objectives achieved.	Consistently positive outcomes when multiple elements are combined: improvements in quality of life, functional status, self-management, satisfaction, and in some cases service use.
13	Ahmed et al. (2022) (38)	Intervention configurations associated with PCC: Contexts (C): training and skills of healthcare professionals, sufficient consultation time, accessibility, supportive policies, team coordination, Information Technology (IT)/e-health; and Mechanisms (M): effective and understandable communication, empathic and respectful attitude, holistic approach, SDM, therapeutic relationship, self-management support.	To make PCC effective in PHC, especially for vulnerable groups/clinical communication, patient participation, care coordination, self-management, accessibility, and equity.	Professional training in communication and PCC; use of clear language and verification of understanding; respectful and empathic attitude; SDM; personalized care plans; sufficient consultation time; coordination and teamwork; organizational accessibility; use of IT/e-health as support. Components act synergistically.	No specific assessment instruments are reported in a standardized way. Results come from multiple studies with heterogeneous instruments (satisfaction, health, self-management, service use).	Includes everything from ongoing clinical practices to interventions with follow-up over time.	Consistently positive outcomes when PCC elements are aligned: greater satisfaction, involvement, concordance, self-management, better psychological and health outcomes, and in some cases better system outcomes (fewer referrals, better coordination). PCC fails when contextual conditions (time, training, accessibility) are absent.
14	Khatri et al. (2023) (39)	People-centred PHC strategies, models, and practices mapped to the five IPCHS components. The interventions are broad configurations of organizational, relational, and community practices.	To make people-centredness effective in PHC/community participation, empathy and communication, leadership and accountability, residential and participatory care models, multimorbidity care, intersectoral coordination, and enabling environments.	Empathy and person-centred communication; user participation in clinical and organizational decisions; participatory and/or residential models of care; interdisciplinary work; health-social integration; organizational leadership and accountability; use of ICT/EHR as support.	Specific instruments are not reported systematically. Studies use heterogeneous measures (user experience, satisfaction, access, continuity, coordination, service utilization).	Not standardized. Includes ongoing practices and medium- and long-term organizational transformations.	Convergent but mostly qualitative/observational outcomes: improved care experience, perceived empathy, participation, continuity, and coordination; in some contexts, reduced hospitalizations and costs. Structural barriers are reported (time, resources, leadership, inequities).
15	Smith et al. (2021) (48)	Case/care management with active patient support to manage multimorbidity; interventions centred on self-management support (structured educational workshops, group workshops, health coaching); medication management and optimization interventions.	To improve outcomes in people with multimorbidity/experience of care; self-management; coordination and continuity; medication use and appropriateness; reduction of fragmentation and treatment burden.	Case/care management, interdisciplinary teams, extended consultations, personalized care plans, telephone follow-up, home visits, group self-management programmes, coaching, professional training, structured medication review, brown-bag reviews, ICT/ Clinical Decision Support Systems (CDSS) support.	Specific instruments are reported, but they are highly heterogeneous: Health-Related Quality of Life (HRQoL), EQ-5D, 12-Item Short Form Health Survey (SF-12), PACIC, CARE, Patient Activation Measure (PAM), Frenchay Activities Index (FAI), Medication Appropriateness Index (MAI), Medication Adherence Report Scale (MARS), Beliefs about Medicines Questionnaire (BMQ), Morisky Medication Adherence Scale(MMAS), among others.	From 6 weeks to 18 months, with most interventions lasting 6–12 months. Follow-up was generally conducted at the end of the intervention; long-term follow-up was uncommon.	Mostly null or small effects on HRQoL and mental health. Modest effects on patient experience, activation, health behaviours, and medication appropriateness in some studies. Low-certainty evidence due to heterogeneity and imprecision.
16	Peart et al. (2018) (44)	Patient navigation interventions, mostly person-based (human navigators) and to a lesser extent process-based (electronic systems).	To connect people without a regular PHC provider to PHC; facilitate a first appointment or effective contact with a provider; reduce barriers to access; improve early continuity of care/access to PHC; emergency department ED–PHC coordination; relational accompaniment; removal of administrative, cultural, and cognitive barriers; patient orientation and education.	Active identification of people without PHC (emergency department, hospitals, community); trained navigators (volunteers, community health workers, lay workers, case managers); bond-building; barrier assessment; direct appointment scheduling; in-person or telephone accompaniment; reminders; education about the system; coordination with clinics and social services.	The primary outcome is behavioural and verifiable: attendance at at least one PHC appointment or effective contact with a provider. Administrative records, EMR, and programme follow-up are used.	Navigation begins at the point of contact (emergency department/community) and ends when the patient schedules or attends the appointment. Follow-up ranges from 14 days to 12–18 months, depending on the study.	Consistent results in terms of improved initial access to PHC. Limited evidence on long-term continuity, clinical outcomes, or cost-effectiveness. Effectiveness appears greater when navigation is relational and personalized.
17	Prajankett and Markaki (2021)(45)	Interventions are grouped into five broad types aligned with the WHO -IPCHS framework: (a) case management and care coordination; (b) empowerment and self-management support interventions; (c) integrated home and community care; (d) quality and safety improvement interventions; (e) use of information technologies and digital support.	To improve quality and continuity of care for older people; respond to complex needs; promote healthy ageing; reduce unnecessary ED use and hospitalizations; strengthen self-management and well-being/individual and community empowerment; care coordination; reorientation of the model toward prevention and promotion; case management; quality and safety improvement; integration of technology and information.	Health education and self-management; SDM; home visits; community programmes; NP–physician co-management; coordination with volunteers and community health workers; clinical review and follow-up; use of IT/EMR; quality-improvement and safety initiatives.	Heterogeneous instruments, depending on each study (quality of life, service utilization, satisfaction, functionality, costs).	From one-off interventions to sustained community-care models.	Convergent findings: improvements in quality of care, empowerment, coordination, access, and, in several studies, reductions in ED visits and hospitalizations.
18	Cooper et al. (2023) (46)	SP interventions in mental health, mediated mainly by link workers (not traditional clinical professionals): an initial relational meeting with a link worker; co-identification of needs and personal goals; and active, supported referral to community resources.	To support well-being and mental health recovery; reduce social isolation; offer non-clinical alternatives; increase sense of control and autonomy/relational accompaniment; referral to community activities; goal-setting; creation of safe and non-stigmatizing social spaces.	One-to-one encounters with link workers; collaborative assessment of needs; active referral to community resources (groups, arts, physical activity); flexible accompaniment; emotional support; continuous review of goals; adaptation to the user's pace and emotional state.	Evaluation is based on qualitative interviews and thematic analysis of user experiences.	Duration depends on user need; no standardized time periods are reported. The intervention may last weeks or months, with variable intensity.	Consistently positive experiential outcomes: greater feelings of support, understanding, and belonging; perceived reduction in loneliness; increased confidence and personal agency. Structural limitations are reported (waiting lists, insufficient community resources).
19	Leonardsen et al. (2023) (41)	Digital interventions in PHC, not standardized or manualized. They include telemedicine, home telemonitoring, digital platforms, apps, structured telephone support, and information-exchange systems.	To improve accessibility and continuity of care; support self-management; increase patient participation; facilitate patient–professional communication; improve understanding of the health situation/access to information and interaction; self-management support; digital communication; remote monitoring; care coordination; situational awareness.	Teleconsultations (telephone, video); digital platforms with personalized information; follow-up apps; telemonitoring (symptoms, vital signs); digital coaching; electronic information exchange. Human contact is complementary.	Clinical questionnaires [e.g., Kansas City Cardiomyopathy Questionnaire (KCCQ), Patient Health Questionnaire (PHQ-9)], locus-of-control scales, motivation scales, System Usability Scale (SUS), sensor records, and *ad hoc* questionnaires.	From short-term pilot interventions to prolonged digital follow-up models.	Improvements in accessibility, participation, and self-management; increased situational awareness. However, risks of depersonalization, digital-literacy barriers, and limitations for the therapeutic relationship are identified when digitalization replaces human contact.
20	Dahm et al. (2022) (51)	Multiple communicative and linguistic strategies to manage/express diagnostic uncertainty in primary care. Two broad types: (1) patient-centred strategies; (2) diagnostic-reasoning strategies; plus linguistic realizations: explicit, implicit, or omission.	To improve patient experience (trust, satisfaction), adherence, and participation in the diagnostic process; reduce diagnostic errors; and communicate risks/plans under uncertainty.	Patient-centred: empathy, re-eliciting the narrative, expectation management, personalized information, safety netting, humour. Reasoning: explaining the diagnostic process, differential diagnosis, information-seeking/consultation. Linguistic realizations: negative statements (“I don't know”), modals (“could”, “probably”), questions/conditionals, intentional vagueness, gestures/gaze.	Experience/satisfaction and trust surveys/questionnaires, experimental vignette studies, audio-video recordings of consultations, measures of adherence and participation; the review applied Quality Assessment Tool for Studies with Diverse Designs (QATSDD) for methodological quality.	Not specified.	Main findings: combining explicit communication of uncertainty with patient-centred strategies improves satisfaction, trust, and the therapeutic relationship; explicitness alone may reduce trust, adherence, and perceived competence; implicit forms show mixed effects; omission may generate frustration when symptoms remain unexplained; explaining the process/reasoning and offering plans increases participation and adherence.
21	Zulman et al. (2020) (49)	Five recommended clinical-presence practices (“Presence 5”), derived from 31 preliminary practices and supported by 73 reviewed interpersonal interventions (mainly training in communication skills, interviewing techniques, mindfulness, clinician–patient relationship approaches, shared agenda-setting, motivational interviewing, and SDM).	To strengthen physician presence in the clinical encounter and the human connection with patients. To improve patient experience (communication, perceived respect, empathy, satisfaction) and professional experience (meaning in work, well-being, reduced burnout). To contribute to the quadruple aim: health outcomes, patient experience, clinician experience, and efficiency/costs. To reduce depersonalization of care in contexts of high technological and administrative burden.	Set of five core practices: (1) Prepare with intention: personalized preparation before the encounter (focused chart review, information from pre-visit questionnaires) and brief centering micro-rituals before entering the consultation (breathing, pausing during handwashing). (2) Listen intently and completely: deliberate use of open non-verbal communication (sitting down, leaning toward the patient, orienting the body toward the patient even when using the electronic health record) and avoiding interruptions, especially during the initial narrative, using silence and sparse but timely questions. (3) Agree on what matters most: initial open-ended questions to elicit the patient's concerns and priorities (“What matters most to you today?”) and construction of a shared agenda, including a final check for pending issues. (4) Connect with the patient's story: exploration of life history and context (social, family, cultural aspects), use of the patient perspective (“walk in their shoes”), and positive reinforcement of efforts and small achievements, with language emphasizing progress and collaboration. (5) Explore emotional cues: attention to verbal and non-verbal emotional cues (tone, facial expression, posture), explicit inquiry into how the patient is experiencing the situation, and empathic responses that name and validate emotions. These practices are supported by formal interventions in communication, empathy, and mindfulness training.	Patient experience/satisfaction questionnaires, measures of professional well-being and burnout, clinical and service-utilization indicators, and some economic aspects are mentioned globally.	Not specified.	Interpersonal interventions frequently improve patient experience (satisfaction, perceived listening, empathy, and quality of communication) and, to a lesser extent, clinical outcomes, professional experience, and costs. The cited evidence indicates that behaviours such as sitting next to the patient, listening without interrupting, agreeing on a shared agenda, and responding to emotions are associated with higher satisfaction, better understanding of information, less distress, and in some cases fewer repeat consultations. Structured training in communication skills, empathy, and mindfulness is linked to lower anxiety, depression, and stress in clinicians, and to reduced emotional burden in patients.
22	Kunneman et al. (2019) (40)	There were patient-directed interventions (*n* = 22), clinician-directed interventions (*n* = 16), and mixed interventions (*n* = 20). Programmes included SDM training for physicians, decision aids/encounter tools, and packages designed to facilitate SDM.	To improve SDM implementation, increase patient participation, enhance empathy/clinician–patient relationship, and reduce distress/decisional regret in some contexts.	Reported components: clinician training in SDM; use of decision aids or encounter tools (e.g., encounter tools, Option Grids); redesign of consultation flow to encourage listening; some studies combined observational and self-report measures.	SDM measures: Shared Decision Making Questionnaire (SDM-Q-9), Observing Patient Involvement (OPTION;12/5), CollaboRATE, Control Preferences Scale (CPS), SDM-Q. Complementary measures: CARE (empathy), DISQ (interpersonal skills), RIAS (interaction analysis), Consumer Assessment of Healthcare Providers and Systems (CAHPS), Four Habits Coding Scheme (4HCS), Consultation Care Measure (CCM).	Frequently not reported or heterogeneous; many trials do not describe intervention duration in detail.	Key synthesized findings: (1) SDM assessments tend to focus on technical aspects; coverage of humanistic aspects is limited (7/192 instrument items were related to humanization). (2) When humanizing aspects were measured, positive correlations were found between empathy and SDM, suggesting an association between relationship/empathy and perceptions of SDM. (3) Interventions that improved technical SDM scores (e.g., use of decision aids) did not always improve relational skills/empathy; in some cases, the tool was used as an educational leaflet rather than as a facilitator of shared conversation. (4) Operational conclusion: evaluating SDM through technical steps may overestimate the presence of humanized SDM.
23	Scalia et al. (2018) (53)	53 “encounter PDA” interventions: 23 in RCTs and 30 in non-randomized studies.	To increase patient knowledge and participation; reduce decisional conflict; improve satisfaction with the process; and assess feasibility/utility and integration into clinical workflows without increasing duration.	Brief paper-based or digital tools (Option Grid, Decision Boards, electronic libraries); presentation of options, benefits/harms, and risk figures; prompts for questions; brief clinician training; organizational strategies (e.g., a nurse introduces the tool before the encounter).	Scales and measures: objective knowledge; Decisional Conflict Scale (DCS); Observer OPTION-12/5; patient satisfaction; trust and anxiety (Spielberger); consultation duration; clinical indicators (Hemoglobin A1c (HbA1c), adherence, bisphosphonate initiation; PHQ-D in depression).	Not specified.	Meta-analysis: increased knowledge; reduced decisional conflict; increased observed participation in SDM; increased satisfaction; no increase in consultation time. Specific clinical effects were inconclusive (diabetes, statins, depression, osteoporosis showed no significant differences). Barriers: perceived time constraints, lack of training, disagreement with content/format; feasibility was greater with training and workflow adjustments.
24	Stacey et al. (2011) (36)	86 “patient decision aid” interventions for 35 clinical decisions (e.g., prostate screening, hormone replacement therapy, breast genetic testing, colon, prenatal, atrial fibrillation, surgeries). Formats: booklet, video, internet, multimedia materials; compared with usual care, general information, or simpler decision aids.	To facilitate active participation and informed decisions; improve knowledge, risk perception, and values clarity; reduce decisional conflict and indecision; and assess effects on choice, adherence, health outcomes, resource use, and consultation times.	Information on options and outcomes; implicit values clarification; information on the clinical problem and outcome probabilities; explicit values exercises, other people's experiences, guidance/steps for deciding and communicating; in some cases, coaching.	International Patient Decision Aid Standards (IPDAS)-related outcomes: knowledge, risk perceptions, values clarity, participation, satisfaction, decisional conflict, proportion undecided, choice, and adherence; plus health outcomes, anxiety/stress, quality of life, costs, and time. Decisional Regret Scale (DRS), State–Trait Anxiety Inventory (STAI), RAND Corporation quality of life questionnaire (RAND-36), among others.	Variable across studies; reported examples include 1 month, 3 months, 6 months, 12 months, and follow-up of approximately 3 years.	Consistent effects in improving knowledge, accuracy of risk perceptions, values clarity, and participation; reduces decisional conflict and the proportion remaining undecided. Effects on choice depend on context (e.g., some surgical or screening decisions). Limited or variable evidence on adherence and health outcomes; generally positive effects on communication but with concerns about possible relational tensions; limited evidence on costs, consultation duration, and litigation.
25	Cáceres Rivera et al. (2024) (59)	Multiple interventions and guidelines in PHC and community settings (telehealth, cardiovascular programmes, intercultural mediation, workforce development; some cost-effective pharmacological interventions).	To improve access to and quality of cardiovascular primary healthcare (CVD PHC) with an ethnic/gender-sensitive approach; enable community empowerment and self-determination; continuity and coordination via telehealth; reduction of inequities and cultural barriers; comprehensive CVD risk assessment in women.	Teleconsultation/videoconferencing, telemonitoring, teleophthalmology, apps and portals; integration of Indigenous health workers (AHW/AGI), transport/logistical supports; intercultural training; multidisciplinary teams; information systems with alerts; telehealth guidelines/regulations; cost-effective strategies (low-dose diuretics, Angiotensin-Converting Enzyme (ACE) inhibitors, polypill; palivizumab in high-risk infants).	Economic evaluations (cost-effectiveness/utilities), utilization and access indicators; acceptability/satisfaction of users and professionals.	Not specified.	Patients: greater acceptability, lower travel burden, and potential clinical improvements reported in the synthesized literature. Professionals: perceived feasibility and utility, with need for cultural competencies. System: telehealth improves accessibility and continuity, with evidence of cost-effectiveness in certain strategies. Barriers: absenteeism, insufficient technical skills, limited local staff, biomedical hegemony. Gender: specific gaps in assessment (lower detection of smoking in women), and a need for policy and intercultural mediation.

## 4 Discussion

This umbrella review synthesizes the available secondary evidence on interventions aimed at promoting humanization in PHC. The findings converge with established frameworks of person-centred care, in which humanization is expressed as care that is respectful of and responsive to patients' preferences, needs, and values, such that these values guide clinical decision-making (60). This convergence is consistent with the reviews included, which emphasize active listening, empathy, a biopsychosocial approach, and participation (37, 49), as well as with conceptual models of patient-centredness that integrate the biopsychosocial perspective, the patient-as-person orientation, and the sharing of power and responsibility, among other key dimensions (61). An important finding of this umbrella review is that many of the included reviews addressed core dimensions of humanization without explicitly using the term humanization in their titles or primary terminology. Instead, the literature frequently employed related concepts such as person-centered care, patient-centered care, SDM, compassionate care, or relationship-centered care. This variability in terminology suggests that humanization is not a universally adopted label in the international literature, despite the presence of interventions that align closely with its underlying principles.

The reviewed evidence reinforces that clinical empathy and micro-level communicative practices are not ancillary, but rather central mechanisms of care quality. External reviews have demonstrated associations between empathy and patient satisfaction, adherence, reductions in anxiety and stress, and patient enablement, with observable clinical effects in certain contexts (62, 63). This pattern helps to explain why the relational interventions synthesized by 37, 49, and 51 tend to yield more robust effects on proximal experiential outcomes than on distal clinical endpoints, which are typically mediated by multiple determinants and require longer time horizons to manifest.

At the same time, this umbrella review shows that humanization cannot be reduced to the clinical encounter alone. A pair of reviews indicate that the very possibility of a humanized relationship depends on organizational conditions such as adequate time, accessibility, continuity, and coordination of care (38, 39). This perspective aligns with the World Health Organization (WHO)'s framework on integrated, people-centred health services, which promotes systems designed around the needs of individuals and communities, with an emphasis on participation and empowerment, as well as enabling environments for care teams and networks (64). In this regard, the Brazilian approach to humanization (26, 42, 43) appears to complement and extend rather than diverge from the international paradigm of people-centered care promoted by the WHO. Whereas international frameworks typically emphasize patient-centered care, SDM, and responsiveness to individual needs, Brazil's National Humanization Policy adopts a broader systemic perspective that frames humanization not only as a feature of the clinical encounter but also as a political and organizational strategy for transforming health systems. In addition to promoting respectful and participatory care, it highlights co-management, democratic governance, shared responsibility among stakeholders, and the well-being and working conditions of health professionals. In this sense, the Brazilian experience broadens the international framework by suggesting that Person-Centered Care also requires the democratization and humanization of the institutional structures through which care is delivered.

At this point, the evidence on multimorbidity is particularly illustrative. Syntheses of RCTs in primary care report modest or null effects on quality of life and clinical outcomes, alongside more consistent signals in care processes and therapeutic appropriateness (48). However, mechanism-oriented reviews suggest that when there is alignment between the intervention and its context (e.g., through clearly defined case management roles, interdisciplinary coordination, and sustained support for self-management) Person-Centered Care can translate into improved patient experiences, continuity, and coping capacity (38, 54). Thus, the challenge lies not only in identifying which intervention components are effective, but in determining the organizational configurations that work for specific populations and contexts.

With regard to SDM, these findings are also consistent with the conceptual literature, which characterizes clinical deliberation as a process involving the exchange of evidence and values within a trusting therapeutic relationship. The review by 40 cautions that instruments used to assess SDM do not always fully capture humanizing dimensions; this aligns with the need, emphasized in patient-centred communication models, to integrate functions such as responding to emotions, managing uncertainty, making decisions, and enabling self-care (65). In practical terms, this suggests that the implementation of decision-making processes should be accompanied by relational training (e.g., empathy, listening, presence) as well as by consultation conditions that allow for genuine deliberation.

In the community and intersectoral domain, SP emerges as a relevant pathway for humanizing primary care, insofar as it addresses social determinants and loneliness/isolation through relational support and connection with community resources (46, 47). This approach aligns with institutional definitions that describe link workers as agents who connect individuals with community supports, co-produce plans centred on their interests, and sustain the process of engagement (66, 67). However, the reviews included indicate that its humanizing potential depends on the capacity and sustainability of community provision, as well as on effective mechanisms of intersectoral coordination, so that referrals do not become procedural formalities without timely or meaningful follow-through.

Finally, the evidence on digital primary care suggests that technologies can enhance access, participation, and self-management, but may also exacerbate inequalities and depersonalize care if they replace human contact (41). From this perspective, the critical challenge lies in designing hybrid and equitable models, incorporating digital literacy, in-person alternatives, and explicit inclusion criteria, so that digital tools function as facilitators of the therapeutic relationship rather than substitutes for it (68, 69).

Taken together, the findings of these reviews suggest that humanizing interventions are not implemented as isolated techniques, but rather as a constellation of relational practices and organizational adaptations that manifest in context-sensitive ways, depending on the clinical problem, the population, and the design of healthcare programmes.

## 5 Conclusions

In summary, this umbrella review shows that humanization in primary healthcare is conceptualized as a multi-level construct integrating relational practices (empathy, presence, communication, and the management of uncertainty), adaptations in organizational structures (continuity, coordination, accessibility, and support for self-management), and community and policy-oriented strategies (participation, equity, a differential approach, and intersectoral integration) (37–39, 49, 59).

The findings suggest that humanization interventions tend to have more consistent effects on proximal indicators of patient experience, participation, and agency (including satisfaction, perceived listening and support, sense of belonging, and reduced access-related friction) whereas effects on distal clinical outcomes are often modest, heterogeneous, or context-dependent, particularly in the presence of multimorbidity (46, 48, 49).

To advance humanization in primary healthcare, strategies should be implemented as integrated programmes, combining training and ongoing support in relational practices with organizational redesign that ensures continuity, adequate consultation time, and accessibility. This also includes the incorporation of linking roles (e.g., navigation and link workers), the strengthening of community networks, and a balanced approach to digitalization that explicitly considers equity and preserves the centrality of the therapeutic relationship. Such directions are consistent with international frameworks on integrated, people-centred health services and with the reviewed evidence on enabling conditions.

One of the main limitations of this umbrella review is the heterogeneity of the topics and interventions that had to be included in order to ensure an adequate number of records in the final selection. As a result, the narrative synthesis could not incorporate sufficient quantitative indicators to establish criteria and comparisons based on the effectiveness and efficiency of the interventions. This, in turn, points to future lines of research that could build on the main findings of these reviews, taking into account the specific primary care contexts in which they have been studied, and generating effectiveness indicators using validated and reliable instruments both for humanization in healthcare and for medium and long-term clinical outcomes.

Beyond generating indicators using validated and reliable instruments, future research should investigate the implementation, sustainability, and comparative effectiveness of humanization interventions in real-world primary care settings, with particular attention to organizational barriers, contextual facilitators, and the conditions required for long-term integration into routine practice. Particular attention should be given to multilevel approaches that integrate relational, organizational, community, and health system components, recognizing that humanization emerges from the interaction of these dimensions rather than from isolated practices. In addition, further work is needed to clarify the conceptual relationships among humanization and related frameworks such as person-centered, people-centered, and relationship-centered care, and to examine how different national and cultural models, including the Brazilian experience, may complement or inform international approaches to person-centered primary health care.
